# Characterization of CD200 Ectodomain Shedding

**DOI:** 10.1371/journal.pone.0152073

**Published:** 2016-04-25

**Authors:** Karrie K. Wong, Fang Zhu, Ismat Khatri, Qiang Huo, David E. Spaner, Reginald M. Gorczynski

**Affiliations:** 1 Institute of Medical Science, University of Toronto, Toronto, ON, Canada; 2 Transplant Research Division, University Health Network & University of Toronto, Toronto, ON, Canada; 3 Division of Molecular and Cellular Biology, Sunnybrook Research Institute, University of Toronto, Toronto, ON, Canada; University Paris Sud, FRANCE

## Abstract

We have previously reported the existence of a soluble form of CD200 (sCD200) in human plasma, and found sCD200 to be elevated in the plasma of Chronic Lymphocytic Leukemia (CLL) patients. CLL cells release CD200 at a constitutive level, which could be attenuated partially by ADAM28 silencing. In this study, we further explored mechanisms of CD200 shedding beyond that of ADAM28, and performed biochemical analysis of sCD200 using materials derived from purified CLL cells and Hek293 cells stably transfected with CD200, and antibodies generated specifically against either the extracellular or cytoplasmic regions of CD200. CD200 shedding was enhanced by PMA stimulation, and the loss of cell surface CD200 could be monitored as a reduction in CD200 cell surface expression by flow cytometry, in parallel with an increase in the detection of sCD200 in the supernatant. Western blot analyses and functional studies using CD200R1 expressing Hek293 cells showed that the shed CD200 detected in CLL and Hek293-hCD200 supernatants lacked the cytoplasmic domain of CD200 but retained the functional extracellular domain required for binding to, and phosphorylation of, CD200R. These data confirms that a functionally active CD200 extracellular moiety can be cleaved from the surface of CD200 expressing cells following ectodomain shedding.

## Introduction

Cancer immunotherapy is limited by the immunosuppressive nature of tumor cells and their microenvironment, often the result of overexpression of immunoregulatory molecules by both tumor cells and tumor-infiltrating effector cells [1]. CD200, a type-I transmembrane molecule with potent immunosuppressive functions through interaction with its receptor, CD200R1, is one such molecule whose expression on lymphoma cells has been shown to dampen their killing by cytotoxic lymphocytes *in vitro* [2].

In addition to expression on the cell surface, many of these immunoregulatory molecules have also been shown to exist in soluble forms [3–5]. The soluble forms of these cell-surface receptors and ligands may be generated by alternative splicing at the mRNA level, as is in the case of CTLA-4, or by mechanisms of ectodomain cleavage by matrix metalloproteases (MMPs) and a disintegrin and metalloprotease (ADAM) family of proteases [6, 7].

Ectodomain shedding is an important mechanism by which proteolytic cleavage of membrane-anchored molecules at the cell surface leads to the release of a soluble form of the molecule into the extracellular microenvironment [6]. Ectodomain shedding plays an important role in the control of immune responses by regulating the release of cytokines, chemokines, cytokine receptors, and many membrane-anchored immunoregulatory molecules [8, 9]. CD23, CD62L, and CD44, which are amongst the molecules shed by lymphocytes, are known to be substrates of ADAM8, ADAM10, ADAM17, and MT1-MMP [10–13]. In Chronic Lymphocyte Leukemia, the detection of a soluble form of the NKG2D ligands, CD23, and CD14 in patient plasma has been shown to have prognostic value [14–18].

We have previously reported the existence of a soluble form of CD200 in CLL plasma using a CD200 sandwich ELISA [19]. Soluble CD200 (sCD200) was detected in normal human plasma and levels were increased in the plasma of CLL patients, where sCD200 levels were correlated with tumor burden, late stage disease, and disease aggressiveness [19]. Correlation analyses looking at the expression levels of 12 ADAM proteases and CD200 shedding by corresponding CLL cells showed ADAM28 plays an important role in the constitutive shedding of CD200 [20].

The studies below were designed to explore other mechanisms of sCD200 release, including involvement of other ADAM proteases/MMPs, and release of CD200 through exosomes/microvesicles. In addition, we also characterized the interaction between sCD200 and CD200R, which is critical for the downstream consequences of the CD200:CD200R axis of immunoregulation.

## Materials and Methods

### Cells

Peripheral blood from CLL patients were collected at routine follow-up visits with written informed consent, and CD19^+^CD5^+^ CLL cells were purified using the RosetteSep human B cell enrichment cocktail (StemCell Technologies, Vancouver, BC) as described previously [2]. Protocols were approved by the Sunnybrook Ethics Review Board. Purified CLL cells were cultured in AIMV medium (Invitrogen, Carlsbad, CA) supplemented with 5x10^-6^M β-mercaptoethanol (2-ME) (Sigma).

Two Hek293 cell-lines permanently transfected with full-length hCD200 (Hek-hCD200) and hCD200R1 (Hek-hR1), respectively, were obtained from Genetec [2]. Cells were grown in the selection medium DMEM-F12 supplemented with 1ug/ml G418 and 10%FBS.

### Reagents and antibodies

Phorbal 12-myristate 13-acetate (PMA) and Ionomycin were purchased from Sigma-Aldrich. PMA was reconstituted to 10mg/ml stocks in DMSO and was further diluted to a working concentration of 40ng/μl in AIMV medium. Imiquimod, a TLR7 agonist, was purchased from LKT Laboratories (St Paul, MN) and reconstituted to 1mg/ml in DMSO. Recombinant TIMP1, TIMP2, TIMP3, and TIMP4 were purchased from R&D Systems and were reconstituted to working concentrations in AIMV medium. The protease inhibitors GM6001 and TAPI-0 were purchased from Calbiochem and reconstituted to 10mM and 1mg/ml stock, respectively, in DMSO.

The monoclonal rat anti-hCD200 antibodies 1B9 and 3G7, and the polyclonal rabbit serum against the extracellular region of CD200 (CD200v+c), were described previously [2]. A polyclonal rabbit serum against the human CD200 receptor (CD200R1) was generated by immunization of rabbits with a fusion protein containing the extracellular region of human CD200R1 with a his-tag at the N-terminal.

Antibodies against CD19 and CD62L used in FACS analyses were purchased from Biolegend. The apoptosis detection kit for staining of Annexin V and 7AAD was purchased from BD Biosciences. The Pan-Cadherin antibody, used as a plasma membrane marker for loading controls in Western blots, was purchased from Abcam.

### Generation of a rabbit polyclonal serum against the cytoplasmic region of CD200

A peptide containing the 19-amino acids of the carboxyl-terminal domain of human CD200 (*KRHRNQDRGELSQGVQKMT*) was synthesized at the Hospital for Sick Children and used to immunize rabbits (Cedarlane Labs, Hornby, Canada):

The resulting antiserum (rabbit anti-CD200 c-tail serum) was confirmed to react with the peptide in an ELISA using pre-immune serum as negative control. To confirm specificity for the cytoplasmic domain of CD200, the anti-serum was tested with cell lysates from Hek-hCD200 cells or cells expressing only the extracellular domain of CD200 on Western blots.

### Exosome depletion

Exosomes were depleted from CLL plasma and tissue culture supernatants by ultracentrifugtion at 100,000g for 70 min, as previously described [21]. In a separate set of experiments, exosomes were precipitated from CLL plasma and/or tissue culture supernatants using the ExoQuickTM (for plasma) or ExoQuick-TCTM (for tissue culture supernatants) exosome precipitation reagent from SBI (Mountain View, CA) according to the manufacturer’s instructions. Plasma and supernatants depleted of exosomes by both methods, as well as exosomes obtained from ExoQuick precipitation, were tested for CD200 in a CD200 sandwich ELISA as described below.

### Constitutive release of CD200 from CLL cells

To assess the effects of TIMPs on the constitutive release of CD200, CLL cells were cultured at 10x10^6^/ml in 400μl volume in 48-well plates immediately after isolation from fresh blood, with or without recombinant TIMPs. Supernatants were collected at 24 and 48 hours and were analyzed in the CD200 sandwich ELISA. All 4 recombinant TIMPs (TIMP1, 2, 3 and 4) were used at a final concentration 450nM. % inhibition of CD200 shedding was calculated using the formula:

$$\%\;Inhibition=(y-x)\times \frac{100}{y}$$

*y* = amount of sCD200 shed (ng/ml) from untreated control cells

*x* = amount of sCD200 shed (ng/ml) from TIMP-treated cells

### Stimulation of CLL cells

Purified fresh CLL cells (10x10^6^/ml) were cultured in 24-well plates at 37°C in 5% CO2 in the presence or absence of the following stimulants: TLR-7 agonist, phorbal 12-myristate 13-acetate (PMA), and Ionomycin. For activation of CLL cells, Imiquimod, PMA, and Ionomycin were used at a final concentration of 3ug/ml, 40ng/ml, and 1μM, respectively. Where the effect of TAPI-0 on PMA-induced shedding was assessed, CLL cells in the same culture conditions were treated with 50μg/ml TAPI-0, with or without PMA stimulation. At 24 and 48-hours after stimulation, cells were harvested and stained for CD200, CD62L, CD19, and 7AAD. % loss of CD200 from the cell surface was calculated from % expression of CD200 on treated cells using that on untreated cells as reference (nominally 100% expression). Tissue culture supernatants were harvested at the same time points to assess for sCD200 concentration in the CD200-sandwich ELISA.

In some PMA-stimulation experiments, membrane proteins were extracted from aliquots of untreated and PMA-stimulated cells using the ProteoJET^™^ Membrane Protein Extraction Kit (Fermentas). Protein concentration in the membrane extracts was determined by Bradford protein assay (Bio-Rad).

### Serum starvation and stimulation of Hek293-hCD200 cells

Hek-hCD200 cells were seeded in 6-well plates at 2x10^6^cells/ml and grown in serum-containing selection medium until ~80% confluency was reached. Supernatants were then removed, and after 2 washings in PBS, 1.5ml of serum-free OPIMEM medium, with or without 40ng/ml PMA, was added per well. Supernatants from untreated and PMA-treated cells were collected at 2, 6, and 24hr time points and sCD200 concentration in each was assessed by ELISA. Supernatants from Hek293-hCD200R1 cells grown under the same conditions were used as negative controls in the ELISA.

### FACS

CD200 cell surface staining was performed using the mAb 3G7 at 0.005μg per sample in 100μl volume, with goat anti-rat IgG-PE (1:100 dilution) as the secondary antibody for detection. For multi-color staining of CD200, CD62L and CD19, fluorochrome-conjugated CD19 and CD62L antibodies were added at predetermined optimal concentrations at the same time as the secondary antibody. All antibody-incubations were performed at 4°C. Single color controls were included in each experiment for compensation purposes, and all samples were analyzed in a Coulter FC500 flow cytometer.

### CD200 ELISA

The CD200-sandwich ELISA was developed for detection of sCD200 in human plasma, and utilized 1B9 as capture antibody and the rabbit anti-hCD200v+c sera as detection antibody. For optimal detection of sCD200 in CLL supernatants, supernatant samples and CD200Fc standards (0.025ng/ml to 2ng/ml), prepared in AIMV medium, were incubated overnight at 4°C on an ELISA plate coated with the capture antibody at 1.25ng/ml and blocked with 5%FBS-PBS. The next day the plate was washed 5 times in PBS+0.01% Tween20, followed by 1-hr incubation with the detection antibody at a 1:2000 dilution at 37°C. The remainder of the steps were performed as described previously [22]. This modified protocol increased sensitivity of the ELISA from 0.05ng/ml, as reported previously, to ~0.01ng/ml.

### Immunoprecipitation of sCD200

For immunoprecipitation of sCD200, 1ml of supernatants from untreated or PMA-treated CLL cells or HekhCD200 cells were incubated overnight with 2μg of 1B9 and 50μl of Protein A/G Agarose bead-suspension (Pierce Biochemicals) at 4°C. The following day, after 2 washes in RIPA buffer containing 1 mM Na_3_VO_4_, the bound immune-complexes were dissociated by boiling in reduced sample buffer containing 0.025% SDS followed by low speed centrifugation. Supernatants containing immune-complexes were loaded directly onto 10% SDS-PAGE gel.

### Western blotting

After transfer onto PVDF membranes, the blots were blocked with 5% milk-TBST for 1 hour at room temperature, and then probed with primary antibodies overnight at 4°C. The following primary antibodies were used in Western blotting experiments: rabbit anti-hCD200v+c serum (1:2000 dilution), rabbit anti-CD200 c-tail serum (1:500 dilution), or rabbit-hCD200R1 serum (1:2000 dilution). Regardless of the primary antibody used, following primary antibody incubation and washings in TBS-T, all blots were probed with goat anti-rabbit IgG-HRP (Jackson) at a 1:10,000 dilution for 45 minutes as secondary antibody. After thorough washing, blots were developed using an ECL Western blot detection kit (GE Healthcare Bio-Sciences).

### CD200R1 phosphorylation by sCD200

Hek293 cells stably transfected with human CD200R1 (HekhR1) were seeded at 80% confluency in 6-well plates, and serum-starved overnight in OPIMEM medium (Invitrogen). The following day cells were washed once with PBS and incubated at 37°C for 15 minutes with one of the conditioned supernatants harvested from stimulation experiments: CLL supernatant from untreated or PMA-treated cultures; and HekhCD200 supernatant (in OPTIMEM). sCD200 levels in all supernatants were assessed in the CD200 sandwich ELISA. AIMV medium, and supernatant from Hek293 cells in OPTIMEM were used as negative controls. For positive control of phosphorylation, a separate well of cells were incubated with 100μM of activated sodium ortho-vanadate (Na_3_VO_4)_ in OPIMEM medium.

After incubation cells were lysed in 500μl of RIPA buffer containing 50mM NaF, 0.2mM Na_3_VO_4_, and protease inhibitors. Following centrifugation at 10,000rpm, supernatants were immunoprecipitated with 1μg of a rabbit polyclonal antibody to the tyrosine phosphorylated cytoplasmic region (a gift from Dr. Karim Berrada, Ingenious Therapetics, NY, USA) [23] in the presence of 50μl of Protein A/G bead suspension overnight at 4°C. Immune complexes were dissociated from Protein A/G beads by boiling in reducing sample buffer. After centrifugation supernatants containing the immune complexes were loaded onto 10% SDS-PAGE gels. Western Blots were performed using the rabbit anti-hCD200R1 serum for detection of CD200R1 in immunoprecipitation (I.P.)-product. Cells treated with activated 100μM activated Na_3_VO_4_ were used as positive controls for phosphorylation.

### Silencing of Adam 10, Adam 17 and Adam 28 in Hek-hCD200 cells

Hek human CD200 cells grown to 70% confluency in 6 well plates were transfected with stealth siRNA silencers for Adam 10, Adam 17 and Adam 28 (Invitrogen, Thermo fisher Scientific) using lipofectamine. Stealth siRNA (250 pmol) was diluted in 250ul OptiMEM. Lipofectamine 2000 (5ul) was diluted in 250ul OptiMEM and incubated for 15 minutes at RT. After 15 minutes incubation, diluted Stealth siRNA was mixed with the diluted lipofectamine (total volume 500ul) gently and incubate for 15 minutes to allow complex formation. The siRNA and lipofectamine complex was added to each well of a 6 well plate containing Hek human CD200 cells. 6h post transfection, cells were kept in culture with or without PMA (30ng/ml)stimulation for 24h. Supernatant from each well was collected and soluble CD200 concentration was measured by ELISA.

### Statistics

Paired and unpaired *t*-tests were used to determine significance between sample means in stimulation experiments and were performed using Prism 5.0 software. Avona analysis was conducted in cases of multiple comparisons before two-sample *t*-tests were performed.

## Results

### CD200 is constitutively shed from the surface of CLL cells

**Fig 1:**
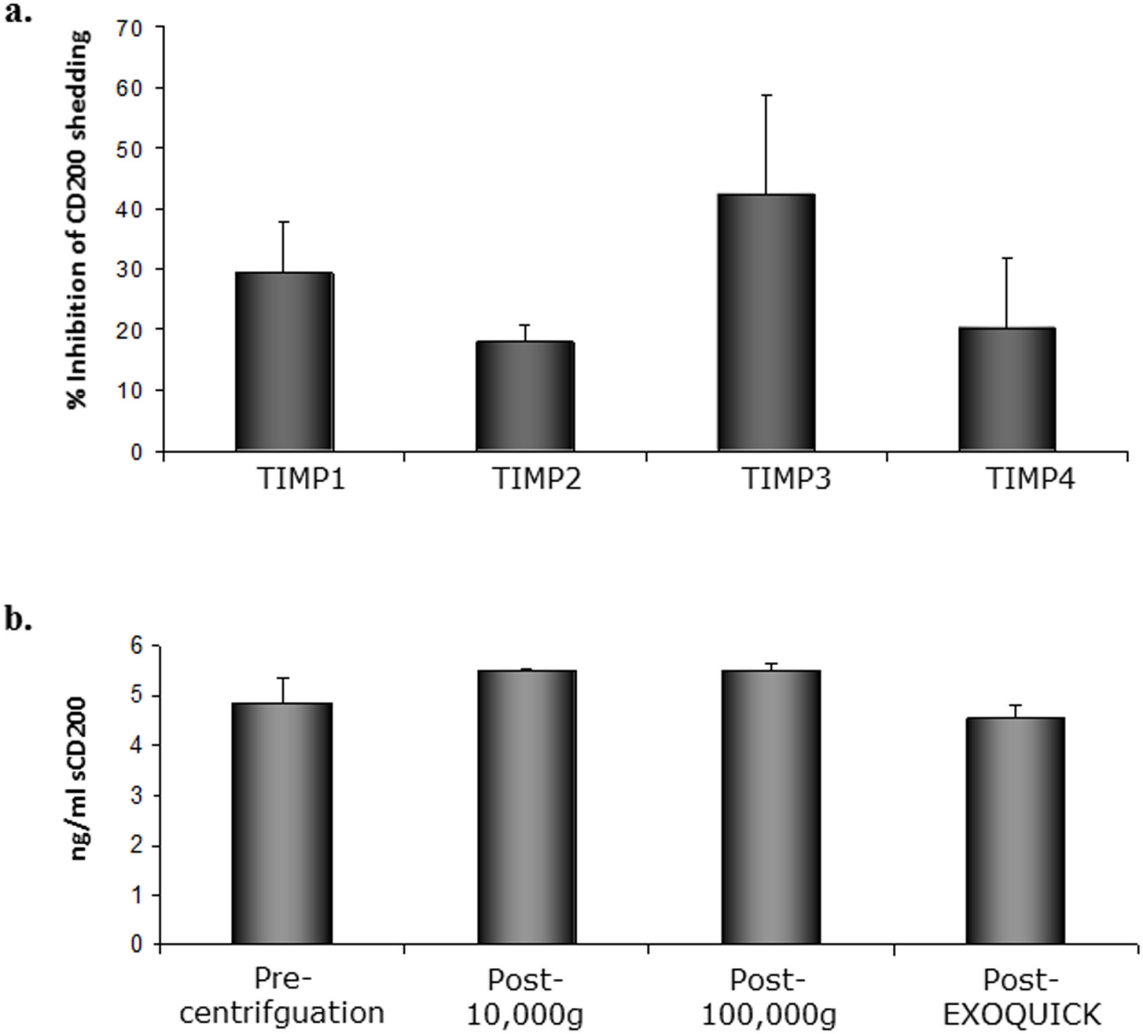
CD200 is constitutively released from CLL cells. a) CLL cells (n = 3) were treated with 450nM of TIMP1, TIMP2, and TIMP3, and TIMP4, and supernatants collected at 48hr. % inhibition of CD200 shedding was calculated with reference to sCD200 levels in supernatants from untreated control cells (varying from 0.1–0.3ng/ml in individual patients). b) CLL plasma was depleted of exosomes/microvesicles using the methodology described. All studies were repeated with three different CLL plasma samples. Data from a representative experiment is shown.

We have previously reported that sCD200 is detectable in CLL tissue culture supernatants, and that the release of sCD200 from CLL cells can be inhibited by the global protease inhibitor GM6001 or by silencing of ADAM28 [19, 20]. To explore whether other MMPs are involved in CD200 shedding, CLL cells were treated with recombinant tissue-inhibitor of metalloproteases (TIMPs), natural inhibitors of ectodomain shedding [24]. Of the four members of the TIMP family (TIMP1-4), each with unique specificity for MMPs and/ ADAM proteases, TIMP3 showed the strongest inhibitory effect on CD200 shedding (Fig 1a, n = 3). At a concentration (450nM), within the range of IC_50_ for the inhibition of CD62L shedding by TIMP3 [25], an average of 42% inhibition of CD200 shedding was observed (Fig 1a). Of the other three TIMPs, TIMP1 was the next most potent inhibitor of CD200 shedding, with a mean 29% inhibition at the same concentration.

Increased release by cancer cells of membrane associated particles such as exosomes or microvesicles containing immunomodulatory molecules plays an important role in cancer biology [26]. To investigate whether sCD200 released by CLL cells can also take place in exosomes/microvesicles, we depleted both forms of membrane particles from CLL plasma by ultracentrifugation and exosome-precipitation using ExoQuick solution. Exosome/microvesicle depletion by either method did not affect sCD200 levels in the plasma (Fig 1b); in addition, CD200 was not detected in exosomes/microvesicles precipitated from the plasma (data not shown), suggesting that these membrane particles are unlikely sources of sCD200.

### CD200 shedding from CLL cells was induced by various stimuli

To investigate whether sCD200 release can be induced by agents known to stimulate ectodomain shedding, CLL cells were stimulated with phorbol myristate acetate (PMA), ionomycin, and Imiquimod, a TLR7 agonist. sCD200 concentrations in the supernatants were assessed at 48 hour.

**Fig 2:**
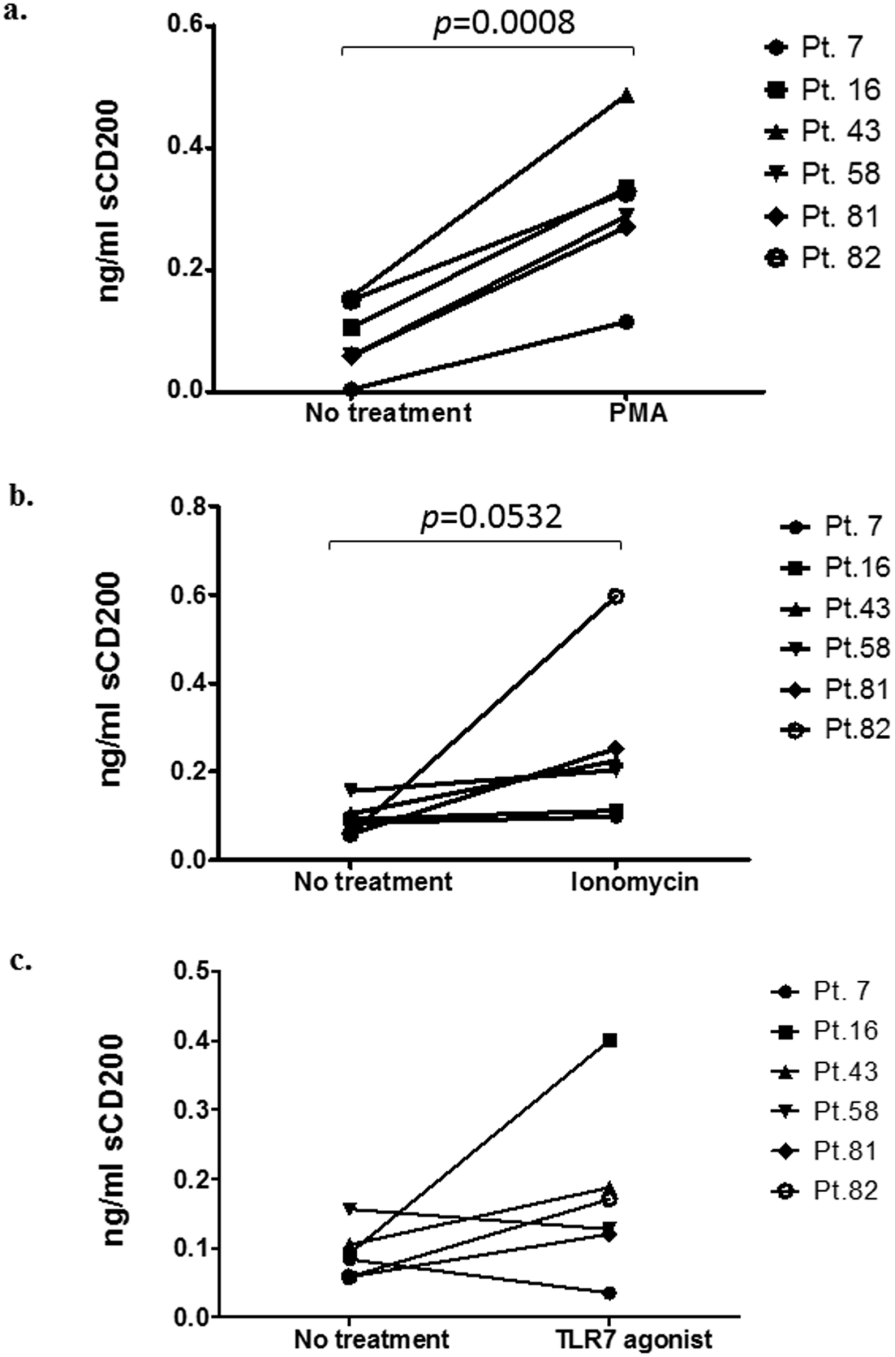
sCD200 is secreted from CLL cells in response to different stimuli. CLL cells (data pooled from 6 individual patients) were cultured in AIMV medium and stimulated with a) 40ng/ml PMA; b) 1μM Ionomycin; and c) 3μg/ml Imiquimod with supernatants collected at 48-hr. At 24-hr, cells were stained for CD62L, CD19, and CD200 expressions by FACS (see Fig 3). *p*-values were obtained from paired t-tests: a) CLL cells from all 6 patients responded to PMA by shedding increased amounts of sCD200 (*p* = 0.0008), but the degree of response to PMA varied amongst patients. b) CLL cells from 4 out of 6 patients showed a modest response to Ionomycin (*p* = 0.0532). 2 out of 6 patients showed no response to Ionomycin treatment. c) CLL cells from 4 out of 6 patients showed a modest response to Imiquimod, although the group mean induction of CD200 shedding was not statistically significant (*p*>0.05).

PMA stimulation increased release of CD200 into the supernatant by CLL cells from all patients tested (*p* = 0.0008, n = 6), although the level of response varied amongst patients (Fig 2a). Stimulation of CLL cells from the same cohort of patients by ionomycin generally produced enhanced shedding *(p* = 0.0532, n = 6), although to a lesser degree than that induced by PMA (Fig 2b). Finally, CLL cells from 4 out of the 6 patients responded to TLR7 stimulation by enhanced release of sCD200 (Fig 2c).

### PMA-induced CD200 shedding was reflected by loss of CD200 from CLL cell surface

**Fig 3:**
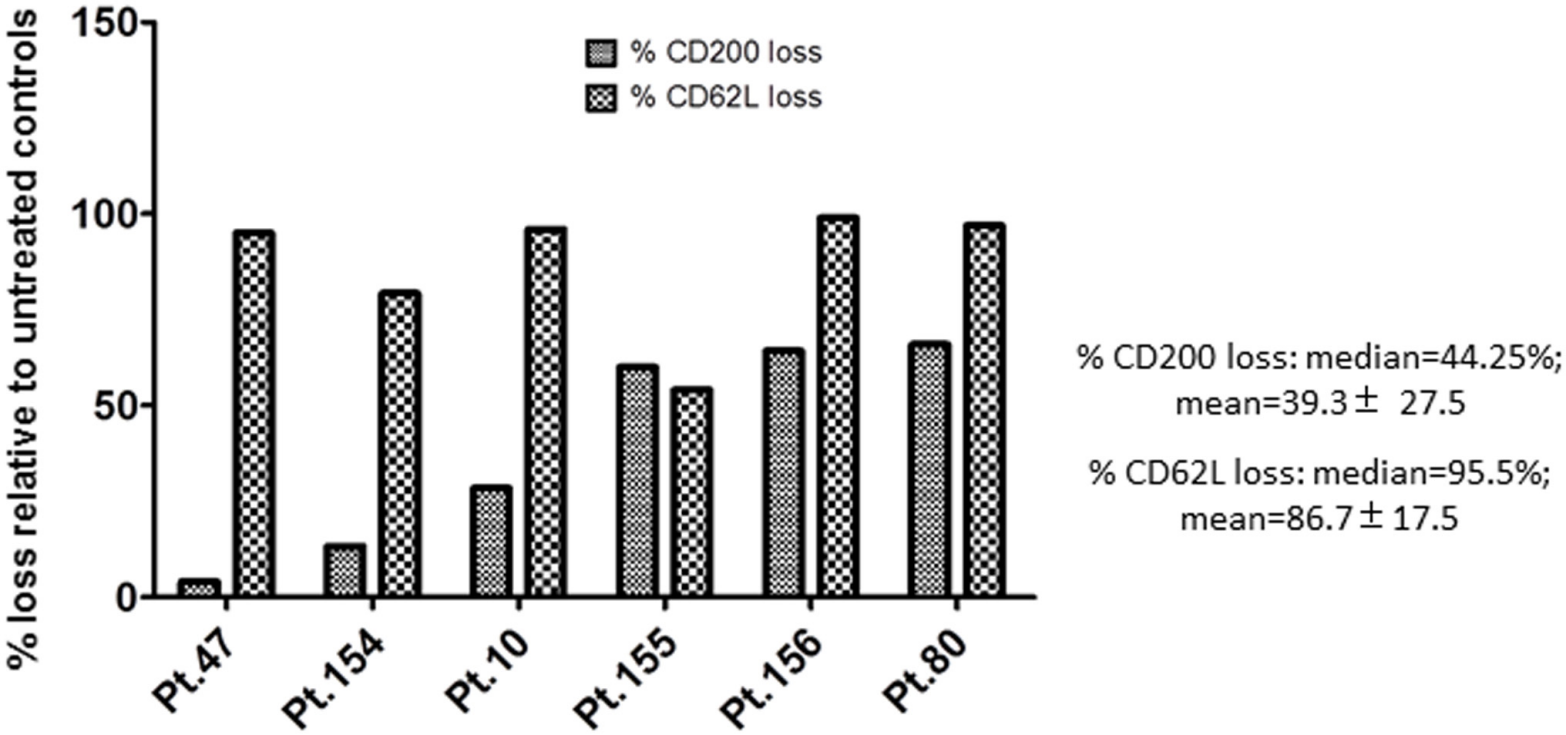
PMA-induced CD200 shedding is reflected as a loss of CD200 expression from the surface of CLL cells. CLL cells from 6 patients were cultured in medium alone, or with 40ng/ml PMA, and harvested at 24 hour to assess for CD200 and CD62L expression. Median and mean percent loss of CD200 and CD62L from the surface of CLL cells from all 6 patients in the cohort were expressed relative to control cultured cells, as determined by FACS. Both CD62L and CD200 expression were normalized to CD19 expression on the same cells.

**Fig 4:**
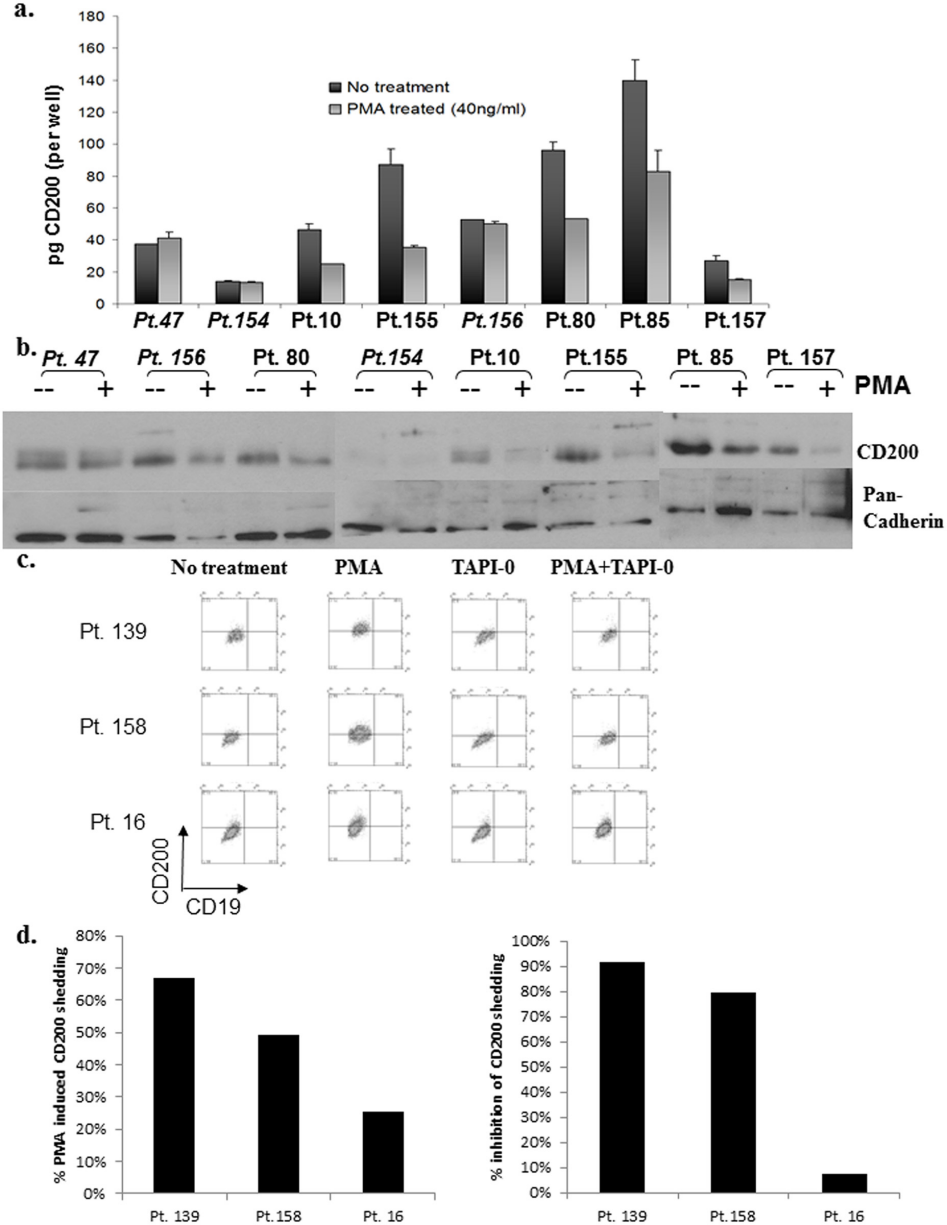
Detection of CD200 in membrane extracts from PMA-treated CLL. Membrane proteins from aliquots of treated and untreated cells from experiments shown in Fig 3 were extracted and analyzed by a) ELISA, and b) Western blotting for CD200. Both methods showed loss of CD200 from the membrane fraction in PMA-treated cells from patients that responded to PMA stimulation as determined by FACS (patients not shedding CD200 in response to PMA as assessed by FACS are highlighted in italic). c) CLL cells from another cohort of patients (n = 3) were cultured in AIMV medium, with or without PMA stimulation, and treated with 50μg/ml of TAPI-0. Cells were harvested at 24-hr and stained for CD200 and CD19. TAPI-0 restored cell surface expression of CD200 on PMA-treated cells from patients 139 and 158 (2 upper panels). CLL cells from patient 16 showed no response (attenuation of PMA-induced sCD200 shedding) to TAPI-0 with PMA causing only a low level of CD200 shedding in this case (bottom panel). d) %PMA-induced shedding, and %inhibition of PMA-induced shedding by TAPI-0 for the three patients shown in panel d).

In lymphocytes, ectodomain shedding of CD62L by ADAM proteases, which is induced upon PMA stimulation, is reflected also in a loss of CD62L from the cell surface by FACS [25]. To explore whether the elevation of sCD200 in the supernatant of PMA treated CLL cells was a function of inducible ectodomain shedding which followed a similar pattern, CLL cells from a different cohort of patients (n = 6) were monitored by FACS for CD200 expression on the cell surface 24h after PMA stimulation (Fig 3; see also S1a–S1c Fig), and by Western blotting and ELISA using membrane extracts harvested from the same cells (Fig 4a and 4b). The shedding of CD62L following PMA stimulation as determined by FACS was used as an independent indicator for inducible ectodomain shedding (Fig 3; see also S1 Fig).

CLL cells from all 6 patients in this cohort responded to PMA stimulation by shedding significant amounts of CD62L from the cell surface, as reflected in an average reduction of 86.7% (±17.5%) in CD62L cell surface staining on PMA-stimulated cells compared to untreated cells. Fig 3, shows the % loss of CD62L and CD200, across all patients, while S1a Fig shows individual data from 3 representative patients. By FACS analysis, CD200 levels were reduced by >20% on the surface of CLL cells from 4 out of the 6 patients following PMA stimulation, with a median loss at 44.2% and an average loss of 39.3% (±27.5%), supporting the hypothesis that CD200 was shed from the cell surface following PMA stimulation. Minimal evidence for apoptosis was seen in all cultures (S2 Fig).

Membrane extracts from PMA-stimulated cells from the 5 patients showing ≥20% CD200 shedding by FACS also had reduced CD200 levels when tested by CD200-ELISA (Fig 4a, 3μg membrane extracts tested, *p* = 0.0391). This reduction in CD200 levels in the membrane extracts after PMA stimulation was further confirmed by Western blotting (Fig 4b). As a control we also examined total cadherin levels in the membrane extracts. Some cadherins, such as E-Cadherin, are known to be substrates for ADAMs (David JM and Rajasekaran AK, 2012, Can Res), although minimal cadherin loss was observed in membrane extracts from PMA stimulated CLL cells, with the exception of patient 156.

As further confirmation that inducible CD200 shedding was mediated by ectodomain cleavage, CLL cells were treated with TAPI-0, a hydroxymate-based inhibitor of metalloproteases [27]. CLL cells from 3 different patients were treated with TAPI-0 with or without PMA stimulation, and CD200 shedding was assessed at 24-hr by FACS. Treatment with TAPI-0 restored CD200 expression on PMA-treated CLL cells from patients 139 and 158 (Fig 4c and 4d). In contrast, confirming the patient-to-patient variability previously observed, PMA induced 25% CD200 shedding in patient 16 with TAPI-0 only minimally effective in inhibiting CD200 shedding (Fig 4d).

Note that sCD200 levels in supernatants measured at the same time for these patients are subject to the confounding effect of a decrease in viability of cells in the presence of TAPI-0. Accordingly, assessing loss of CD200 from the cell surface (as here) by gating on live cells represents a superior characterization of the CD200 shedding process in the presence of TAPI-0.

### CD200 shedding in the epithelial Hek293-hCD200 cells

**Fig 5:**
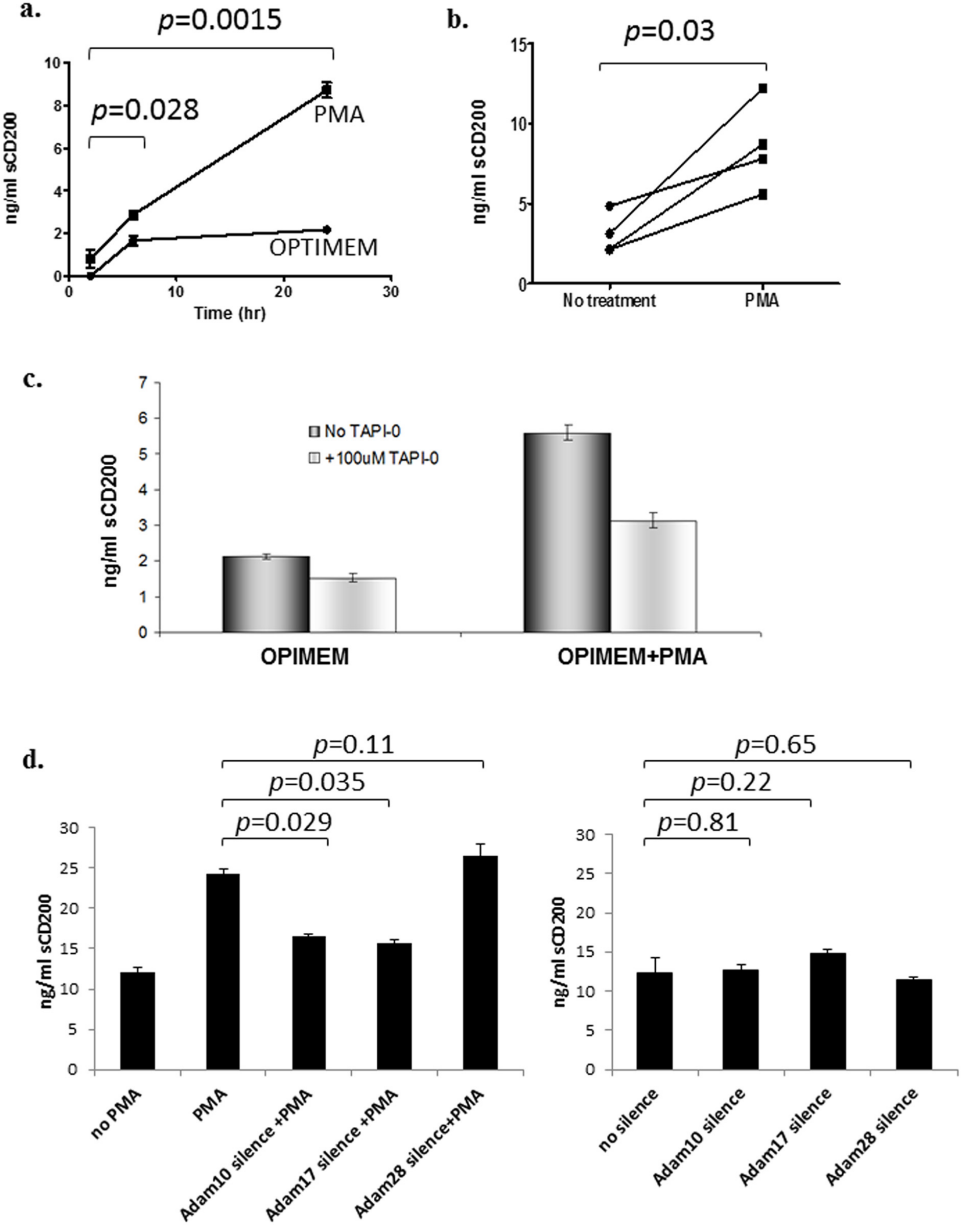
Constitutive and inducible shedding of CD200 in Hek-hCD200 cells. Hek-hCD200 cells were grown in serum-free OPIMEM medium with or without 40ng/ml PMA stimulation. Supernatants were collected at different time points and assessed for sCD200 concentration by ELISA. a) sCD200 was detectable in supernatants from untreated cells at 6-hr after serum starvation with sCD200 concentrations remaining relatively stable to 24-hr. PMA-treated cells released two-fold more sCD200 at 6-hr (*p* = 0.0278) and by 24-hr showed 4-fold higher concentration of sCD200 (*p* = 0.0015). Data pooled from 4 studies are shown. b) Hek-hCD200 consistently shed increased amounts of sCD200 in response to PMA compared with control cultured cells ("no tretament") as detected in 24-hr supernatants in 4 independent studies (*p* = 0.03, paired t-test). c) Serum-starved Hek-hCD200 cells, with or without PMA stimulation, were treated with 50μg/ml TAPI-0, and supernatants were harvested at 24-hr. TAPI-0 inhibited PMA-induced CD200 shedding by Hek-hCD200 cells (~45%), with minimal effect on constitutive shedding (OPIMEM). All experiments were repeated 3 times. d) Hek-hCD200 cells, transfected with ADAM 17, ADAM10, and ADAM28 stealth siRNA, were kept in culture with or without the presence of PMA(30ng/ml), and supernatants were harvested at 24-hr. Both ADAM17 and ADAM10 silencers independently attenuated PMA induced sCD200 shedding from Hek-hCD200 cells (*p* = 0.029, *p* = 0.035 respectively). However, neither ADAM10 or ADAM17 silencers showed modulated constitutive CD200 shedding (*p* = 0.81, *p* = 0.22 respectively). ADAM28 silencers, as a negative control, did notattenuate CD200 shedding with or without PMA stimulation.

Given that tissue CD200 expression is relatively ubiquitous, and its overexpression has been reported on a number of solid tumors [28, 29], we next investigated whether CD200 shedding occurred in cells of epithelial origin using a Hek293 cell line stably transfected with full-length human CD200 (Hek-hCD200) [2]. Like CLL cells, serum-starved Hek-hCD200 cells released CD200 constitutively, with detectable levels as early as 6-hrs after serum starvation (Fig 5a, *p* = 0.0278, data from a typical study are shown). Importantly, Hek-hCD200 cells also responded to PMA-stimulation by shedding increased amounts of sCD200, with up to 3-fold more sCD200 present in supernatants from PMA-stimulated cells compared to supernatants from untreated cells by 24hr (Fig 5b; *p* = 0.03, paired-*t* test). The inducible, but not constitutive, release of CD200 by Hek-hCD200 cells was inhibited by TAPI-0 (Fig 5c). Hek-hCD200 cells express ADAM 10 and ADAM 17 constitutively, but not ADAM28 (unpublished data). When transfected with ADAM 10 or ADAM17 stealth siRNA, Hek-hCD200 cells shed less sCD200 (*p* = 0.029, *p* = 0.035 respectively) in the supernatant after PMA (40ng/ml) stimulation (Fig 5d). However, transfection with ADAM10 or ADAM17 stealth siRNA failed to attenuate constitutive CD200 shedding in Hek-hCD200 cells (*p* = 0.81, *p* = 0.22 respectively). ADAM28 silencing, as a negative control, produced no effect on PMA-induced or constitutive CD200 shedding in Hek-hCD200 cells(Fig 5d).

### sCD200 released by CLL and Hek293-hCD200 cells did not contain the cytoplasmic domain of CD200

**Fig 6:**
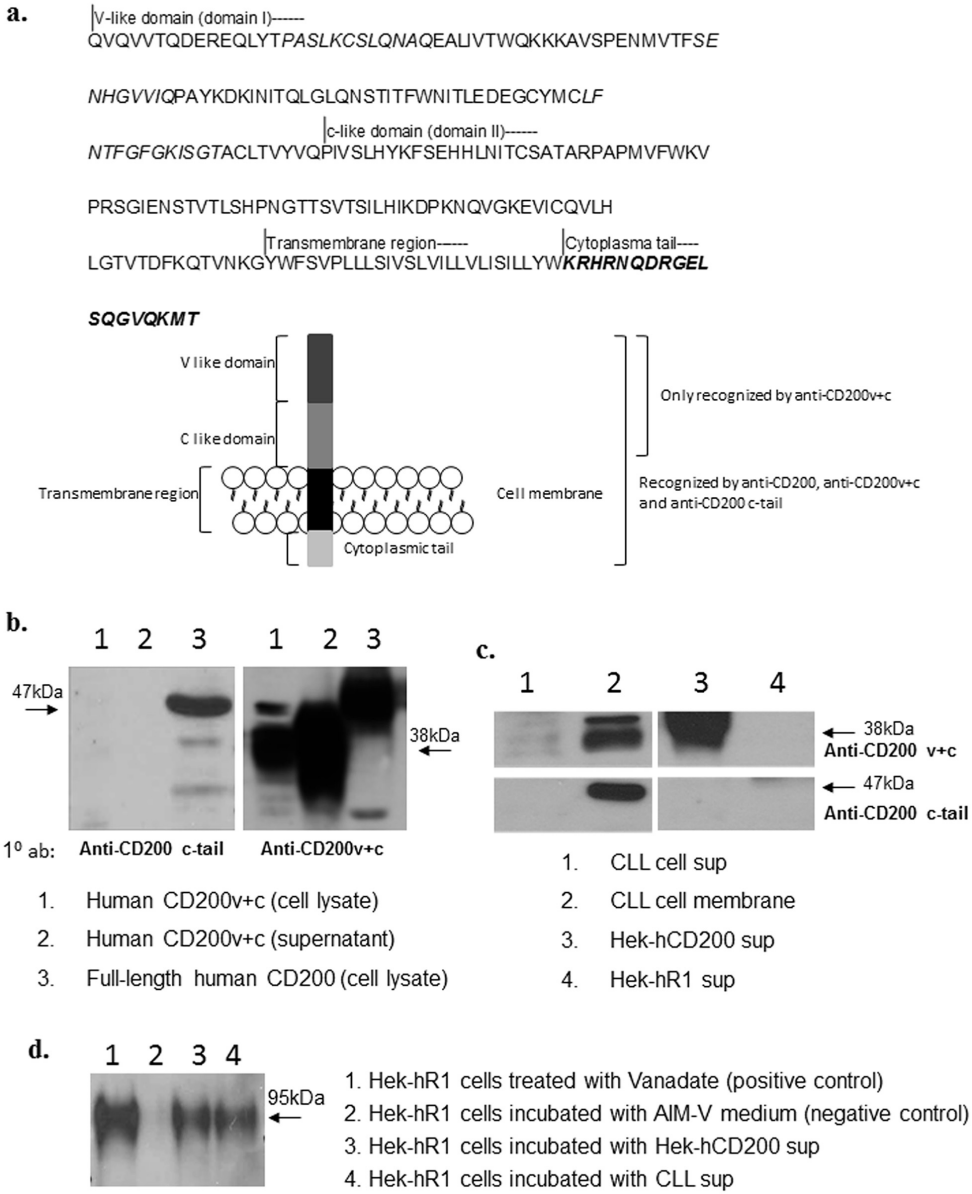
Absence of epitopes associated with the cytoplasmic domain of CD200 in sCD200 and functional properties of sCD200. a) Amino acid sequence of full-length human CD200. The peptide sequence used for generation of a rabbit anti-CD200 cytoplasmic-tail antibody is highlighted in bold. The diagram showed proteins detected in Fig 6b-d, and the antibodies used to detect them. b) Characterization of rabbit anti-CD200 c-tail serum; the antiserum recognized lysates from Hek-hCD200 cells (lane 3), which expressed full-length CD200, but not lysates or supernatant from Hek293 cells transfected with hCD200v+c (lane 1 and lane 2), indicating specificity for the cytoplasmic domain of CD200. However, they were all recognized by anti-CD200v+c antibodies. c) Membrane extracts from CLL cells were recognized by both rabbit anti-hCD200v+c serum and rabbit anti-CD200 c-tail serum (lanes 2). sCD200 immunoprecipitated from CLL (lanes 1) and Hek-hCD200 (lane 3) supernatants was recognized only by rabbit anti-hCD200v+c serum, but not by rabbit anti-CD200 c-tail serum, indicating that sCD200 released from both cell types lacked the cytoplasmic domain of CD200. Immunoprecipitates from Hek-CD200R1 transfected cells were, as expected, not recognized by either anti-CD200v+c or anti-CD200 c-tail serum (lane 4). d) Hek-CD200R1 cells were immunoprecipitated with anti-phosphotyrosine antibody specific for the phosphorylated tyrosine in the CD200R1 tail [23] following incubation with sCD200-containing CLL and Hek-hCD200 supernatants. Products were subsequently run on 10% SDS-PAGE gel and probed with the rabbit anti-hCD200R1 serum. CD200R1 was phosphorylated by both CLL (lane 4) and Hek-hCD200 (lane 3) supernatants, but not AIMV medium (lane 2), consistent with the hypothesis that sCD200 was capable of binding to, and causing phosphorylation of CD200R1.

sCD200 released by CLL and Hek293-hCD200 cells contained extracellular domains of CD200, as illustrated by its recognition by two antibodies raised against the extracellular regions of CD200: 1B9 and the polyclonal rabbit anti-hCD200v+c serum. For specific recognition of the cytoplasmic region of CD200, we generated a polyclonal rabbit anti-serum against a peptide containing the 19 amino acids that made up its cytoplasmic tail (Fig 6a), which reacted only with full-length CD200, but not CD200v+c, a peptide containing only the extracellular region of CD200 (referred to as anti-c-tail serum, Fig 6b).

We confirmed that sCD200 immunoprecipitated from PMA-stimulated CLL or serum-starved Hek-hCD200 supernatants using 1B9 antibody was recognized only by anti-serum against CD200v+c (as a ~47kd band, Fig 6c, upper panel), but not by the anti-c-tail serum (Fig 6c, lower panel), indicating that sCD200 from both sources lacked the cytoplasmic tail. Note that the band intensities of the immunoprecipitated products from Hek-hCD200 supernatant and CLL supernatant differed substantially, but were consistent with quantitation given by ELISAs (Fig 2a).

### sCD200 in CLL supernatant was capable of interacting with hCD200R1

Phosphorylation of an ITIM-like motif in the cytoplasmic region of CD200R1 following CD200 binding is crucial to signal transmission to mediate the downstream functions which characterize the CD200:CD200R1 axis of immunoregulation [30]. To determine whether sCD200 released from CLL and Hek-hCD200 cells was capable of binding and phosphorylating CD200R1 on Hek293-CD200R cells, we probed for the presence of CD200R1 in protein immunoprecipitated with an anti-phospho-tyrosine antibody specific for the phosphorylated tyrosine in the CD200R1 tail (see Materials and Methods) following incubation of the cells with sCD200-containing supernatants. CD200R1 bands were detected in the immunoprecipitate from cells incubated with CLL supernatants (Fig 6d, lane 4) or Hek-hCD200 supernatant (Fig 6d, lane 3), but not in cells stimulated with sCD200^-^ control supernatants (Fig 6d, lane 2)-data shown are representative of one of 3 similar studies.

## Discussion

We previously reported that constitutive release of sCD200 from CLL cells was partially reduced by GM6001, a global inhibitor of metalloproteases and silencing of ADAM28, supporting the hypothesis that sCD200 is the product of ectodomain shedding [19, 20]. Indeed, analysis of the human CD200 gene sequence suggests that, unlike CTLA-4, alternative splicing is an unlikely mechanism responsible for the production of sCD200, as the only reported isoform of CD200 is a truncated form lacking amino acids at the N-terminal [31]. In this study we have further explored mechanisms of CD200 release, and provide conclusive evidence for ectodomain shedding of CD200, which can occur in CD200-expressing cells of epithelial and lymphocyte origin, under both resting and activated conditions.

Although we reported that silencing of ADAM28 partially attenuated CD200 shedding in CLL cells, it is evident from data in this manuscript that sCD200 release is also sensitive to inhibition by the tissue inhibitor of metalloproteases (TIMPs). Of the three major TIMPs (TIMP1-3), TIMP3 has been documented extensively to inhibit shedding known to be mediated by ADAM proteases, including CD62L, IL6 receptor, and Syndecans, in addition to inhibiting MMPs [25, 32, 33]. TIMP1 is known to inhibit MMPs, and has also been shown to inhibit ADAM10 *in vitro* [24, 34]. TIMP2, on the other hand, inhibits only MMPs with no effect on ADAM proteases [24]. The observation that TIMP1 and TIMP3 treatment resulted in the strongest inhibition of CD200 shedding suggests involvement of ADAM proteases as well as MMPs in this process.

sCD200 release from CLL cells, like that of other well-characterized substrates of sheddases such as CD62L and CD44, could be further induced by external stimuli, including PMA and ionomycin [12, 35], through activation of intracellular 2^nd^ messenger systems such as PKC or intracellular Ca^2+^ pathways [36]. Of the three stimuli of ectodomain shedding tested in this study, PMA, a potent activator of PKC particularly known for its ability to induce shedding by ADAM17, was the most effective in enhancing CD200 shedding [37, 38]. Ionomycin, a calcium ionophore that induces different ADAM proteases, most noticeably ADAM10, also enhanced shedding of CD200, though the strength of the response was generally less than that observed with PMA stimulation [39–41]. The heterogeneity in response to the different shedding stimuli by CLL cells from different patients is consistent with heterogeneity in constitutive shedding of CD200 and the variability observed in the responses of CLL cells to inhibition by TIMPs. This may in turn reflect the different expression and/or activity levels of MMPs and ADAMs in CLL cells from each patient.

A number of physiological stimuli induce ectodomain shedding in a variety of cell types. LPS, for example, induces ADAM17 activity through TRIF adaptor signalling that involves downstream activation of NADPH oxidase and PKCδ in phagocytes [42]. In the study described above we found that Imiquimod, a TLR7 agonist, induces CD200 shedding in some, but not all, patients. The response of CLL cells to other physiologically relevant stimuli, including cytokines, remains to be explored. In patients, the proliferating pool of CLL cells are known to reside in “proliferation centers” in association with a non-CLL microenvironment which provides additional stimuli either through soluble factors or cell-cell contact that are important in sustaining CLL survival and growth through multiple pathways [43]. Given the abundance of external stimuli in these microenvironments, the inherent ability of CLL cells to shed CD200 in response to different stimuli, in addition to their ability to shed CD200 constitutively, could contribute significantly to circulating sCD200 in CLL plasma and to the local effects of CD200 in these environments.

We also explored CD200 shedding in non-CLL cells. In preliminary studies, normal B cells, which expressed CD200 constitutively at low levels, also respond to PMA by shedding CD200 (S2 Fig). CD200 shedding was also observed in the epithelial Hek293 cell line stably transfected with CD200. The latter cells shed CD200 constitutively when cultured in serum-free conditions and, like CLL cells, also responded to PMA by shedding increased amount of sCD200. Note that while sCD200 was detectable after 4–6 hours stimulation with PMA, given the limits of detection in the assay used, and the variability in sCD200 release amongst different CLL patients, all the stimulation experiments are done within 24 hr. Interestingly, TAPI-0 did not inhibit constitutive shedding of CD200 by Hek293 cells in serum-free conditions, suggesting the mechanism(s) of constitutive shedding is (are) distinct from PMA-induced CD200 shedding in Hek293 cells. We also investigated the role of ADAM10 and ADAM17 in PMA-induced sCD200 shedding of Hek293 cells using stealth siRNA silencers and showed that both ADAM silencers inhibited PMA-induced but not constitutive sCD200 shedding, consistent with the results showed in the TAPI-0 inhibition experiment mentioned above. Residual shedding which occurred after silencing individual ADAMs we presume reflects the redundancy in activity of other metalloproteases.

Importantly, biochemical analyses reported here demonstrated that sCD200 contains only the extracellular regions of CD200 and lacks a cytoplasmic tail, a feature that is consistent with other products of ectodomain shedding. The exact cleavage site(s) on CD200 remains to be elucidated. The recognition of cleavage substrates by sheddases is thought to involve conformational shapes rather than specific peptide sequences [44, 45]. Glycosylation has also been show to modulate both constitutive and induced ectodomain shedding, and its role in CD200 shedding remains to be explored [46, 47].

Regardless of the mechanism(s) of CD200 shedding, a functional significance for sCD200 was demonstrated by the ability of sCD200 to bind and phosphorylate CD200R1, the major receptor responsible for mediating the downstream immunoregulatory functions of CD200 [30], using an anti-phosphotyrosine antibody specific for the active tyrosine residue in the CD200R1 tail [23]. With documentation of the functional ability of sCD200 to interact with CD200R, the existence of sCD200 in plasma may have important downstream physiological consequences and could play a role in different pathological conditions. It remains unknown whether sCD200 activation of the CD200-CD200R1 pathway occurs with the same stoichiometry/efficiency as when membrane CD200 interacts with CD200R1, and this issue is currently under investigation in our chronic lymphocytic leukemia model. It is also worthy of note that we previously reported on a truncated form of CD200 (CD200tr) which acts as an antagonist of CD200 in mice [31]. Similar variants of CD200 have also been described in humans [48], though to our knowledge, no antibodies, including those used in the studies above (RMG-unpublished), distinguish these variants in human. The data in this manuscript have not explored the relative expression of full-length or truncated CD200 in either membrane-bound or soluble form from CLL cells, although the functional inhibition previously reported for sCD200 [19] suggests the material studied is predominantly the full-length material.

Given the immunoregulatory properties of CD200, and sCD200, insights into mechanisms of CD200 shedding may provide a rationale for therapeutic targeting of this pathway and/or the use of sCD200 as a biomarker [49]. Recent data from our laboratory suggests that sCD200 can also be detected in the serum of breast cancer and colonic cancer patients (unpublished data), consistent with the growing evidence that CD200 itself is reported to be overexpressed in a number of human cancers.

## Supporting Information

S1 Fig PMA-induced CD200 shedding is reflected as a loss of CD200 expression from the surface of CLL cells. (TIF)

S2 Fig PMA induced CD200 shedding in B cells from healthy donor. (TIF)
