# Supplementary figures and images for: Characterization of CD200 Ectodomain Shedding
Source: PLoS One. 2016 Apr 25;11(4):e0152073. doi: 10.1371/journal.pone.0152073 (PMC4844103; doi:10.1371/journal.pone.0152073)

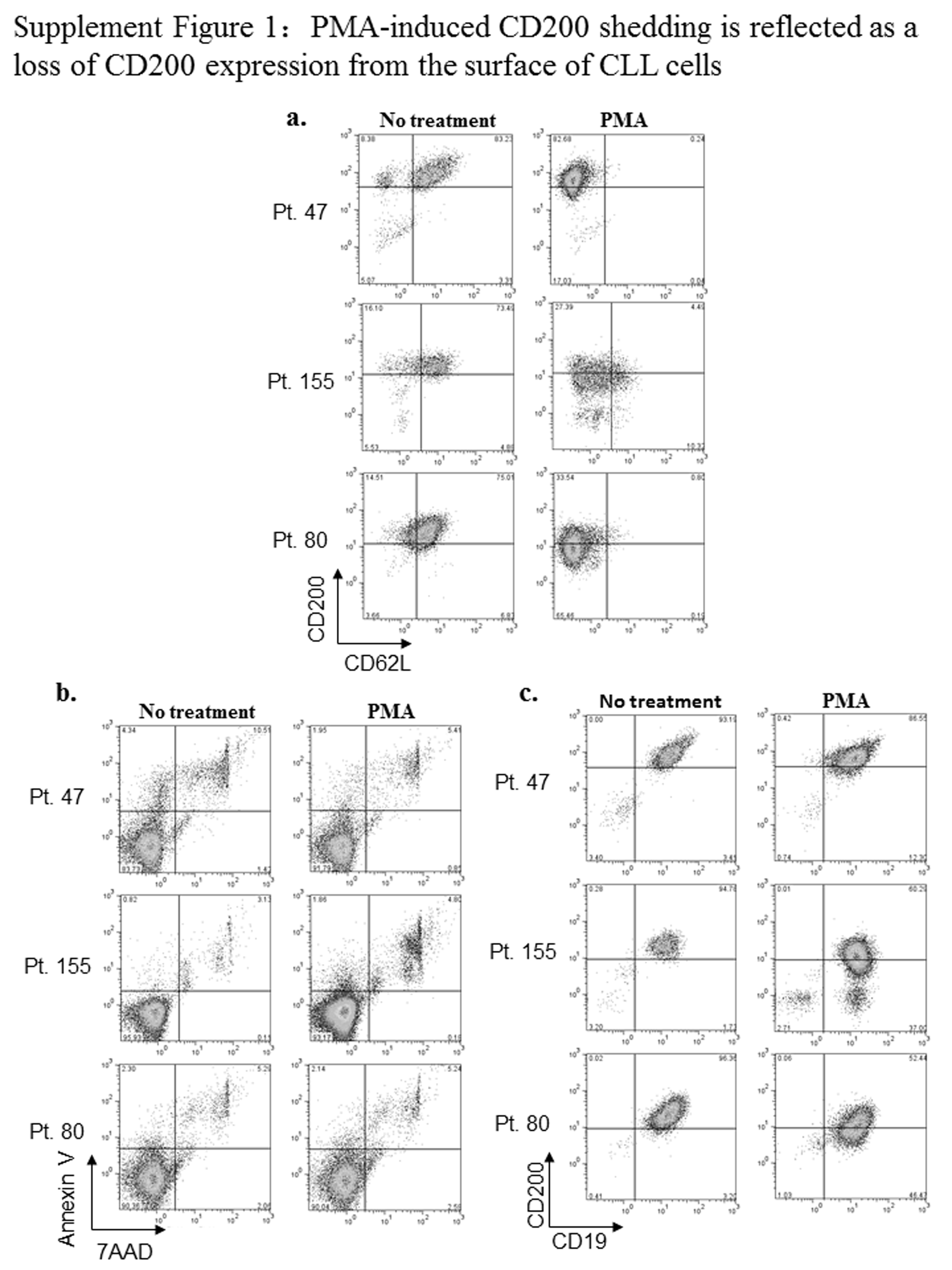

Supplement: S1 Fig — (TIF) [file pone.0152073.s001.TIF]

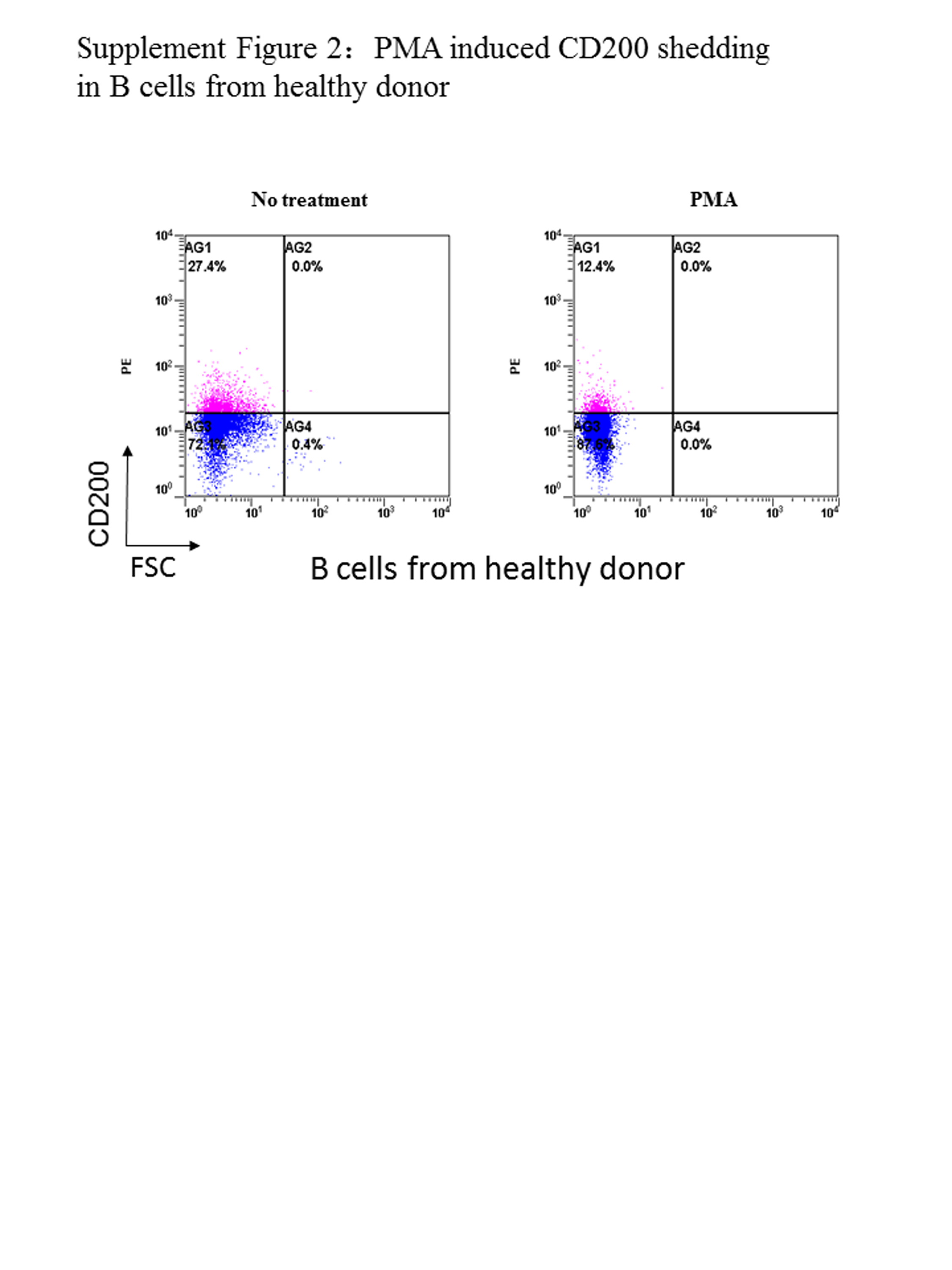

Supplement: S2 Fig — (TIF) [file pone.0152073.s002.TIF]
